# Nebulised Chinese herbal medicine for paediatric pneumonia: a meta-analysis

**DOI:** 10.3389/fped.2026.1777233

**Published:** 2026-07-03

**Authors:** Jing Shen, Yousong Li, Mengling Wei, Tiantian Qin

**Affiliations:** Department of Traditional Chinese Medicine, Shanxi Bethune Hospital, Shanxi Academy of Medical Sciences, Third Hospital of Shanxi Medical University, Tongji Shanxi Hospital, Taiyuan, Shanxi, China

**Keywords:** Chinese herbal, drugs, meta-analysis, nebulisers and vaporisers, pneumonia, randomised controlled trial

## Abstract

**Objective:**

This study aimed to systematically evaluate the clinical efficacy and safety of nebulised Chinese herbal medicine as an adjunctive therapy for paediatric pneumonia.

**Methods:**

A comprehensive search was conducted in the PubMed, Cochrane Library, Web of Science, Embase, China National Knowledge Infrastructure, Wanfang, VIP and Chinese Biomedical Literature electronic databases between inception and 1 November 2025. Randomised controlled trials (RCTs) investigating nebulised Chinese herbal medicine as an adjunctive treatment for paediatric pneumonia were included. Two reviewers independently performed literature selection and data extraction and assessed the methodological quality using the Cochrane risk-of-bias tool. Meta-analysis was performed using Stata 17.0 software, and publication bias was assessed using RevMan 5.4.

**Results:**

Seventeen RCTs involving 1,764 patients were included. The meta-analysis showed that, compared with conventional Western therapy alone, the combination with nebulised Chinese herbal medicine significantly improved the total clinical effective rate [odds ratio = 3.24, 95% CI: (2.24, 4.68), *p* < 0.001] and significantly shortened the resolution time of respiratory symptoms [standardised mean difference (SMD) = −1.10, 95% CI: (−1.56, −0.64), *p* < 0.001], fever resolution time [SMD = −1.01, 95% CI: (−1.59, −0.43), *p* = 0.0006] and hospital stay duration [SMD = −1.06, 95% CI: (−1.38, −0.73), *p* < 0.001].

**Conclusion:**

Current evidence suggests that nebulised Chinese herbal medicine combined with conventional Western therapy can significantly improve clinical efficacy, accelerate symptom resolution and shorten the disease course in children with pneumonia. However, due to methodological limitations in the included studies, these findings warrant further validation in larger, rigorously designed trials.

## 1 Introduction

Pneumonia remains a leading cause of morbidity and mortality in the paediatric population worldwide, posing a significant threat to global child health, particularly among children aged <5 years (1, 2). The management of paediatric community-acquired pneumonia (CAP) often involves antimicrobial therapy; however, current guidelines recommend that antibiotics are not routinely administered to preschool-aged children, as viral pathogens [e.g., respiratory syncytial virus (RSV)] are responsible for the majority of cases (3, 4). Indeed, national surveillance data from China have shown that among hospitalised children with severe CAP, RSV (21.30%) is the most frequently detected pathogen, followed by *Streptococcus pneumoniae* (12.61%) (2). Even when antibiotics are indicated, the escalating challenge of antimicrobial resistance and the potential for adverse effects necessitate the exploration of effective adjuvant treatments that can accelerate symptom resolution, reduce disease burden and potentially minimise unnecessary antibiotic exposure (5, 6).

In this context, traditional Chinese medicine (TCM) offers a rich therapeutic heritage. Over the past decades, several clinical studies and systematic reviews have explored the role of TCM as an adjunctive treatment for paediatric pneumonia. For instance, a systematic review by Guo et al. (7) concluded that oral or injectable TCM formulations, when added to conventional therapy, could improve clinical efficacy and shorten fever duration in children with CAP (7). However, this and other reviews have largely focused on systemic administration routes (e.g., oral decoctions, intravenous injections), which may be associated with variable bioavailability, systemic side effects and lower patient compliance, especially in young children. More importantly, none of these prior reviews has specifically addressed the nebulised route of TCM administration, which offers distinct pharmacological and clinical advantages.

Nebulised inhalation, as a targeted drug delivery system, enables direct deposition of therapeutic agents onto the bronchial mucosa and alveolar surfaces, thereby achieving high local drug concentrations, reducing systemic exposure and enhancing bioavailability (8, 9). This route is particularly attractive in paediatric respiratory infections, where rapid onset of action and avoidance of systemic side effects are critical. Despite the increasing clinical use of nebulised TCM in many regions, the existing evidence has never been systematically quantified or compared with that of other administration routes or placebo controls. Individual randomised controlled trials (RCTs) on this topic suffer from small sample sizes, variable methodological quality and inconsistent outcome measures, leaving clinicians without a robust evidence base.

Unlike prior systematic reviews that focused on oral or injectable TCM (7) or those that evaluated nebulised non-TCM agents, such as saline or bronchodilators (10–12), the present study is the first meta-analysis dedicated exclusively to nebulised Chinese herbal medicine as an adjunctive therapy for paediatric pneumonia. Although previous reviews have reported the general positive effects of TCM, they did not isolate the inhalation route, nor did they account for the potential non-specific therapeutic effects of nebulisation itself, such as enhanced mucociliary clearance and airway hydration (13, 14). Moreover, earlier meta-analyses typically did not quantify the magnitude of effect on discrete clinical endpoints, such as respiratory symptom resolution time, fever clearance and hospital stay duration, nor did they systematically assess the confounding role of nebulisation as a procedural intervention.

The novelty of the present study lies in several interconnected aspects. First, to our knowledge, this is the first meta-analysis dedicated exclusively to nebulised Chinese herbal medicine as an adjunctive therapy for paediatric pneumonia, thereby filling a major gap in the literature. Second, beyond simply aggregating data on total clinical efficacy, we quantify effect sizes on clinically meaningful time-to-event outcomes, such as respiratory symptom resolution, fever clearance and hospital discharge, offering estimates that are directly interpretable for clinical practice. Third, and most importantly, this review critically identifies and discusses a major methodological confounder that has been systematically overlooked in previous TCM meta-analyses—namely, the absence of a placebo nebulisation control in all included studies. By highlighting this limitation, we set a new methodological standard for future research and draw attention to the need for placebo-controlled designs that can isolate the specific pharmacological effects of herbal ingredients from the well-documented non-specific muco-active effects of nebulisation itself.

The primary purpose of this systematic review and meta-analysis is therefore to critically evaluate the clinical efficacy and safety of nebulised Chinese herbal medicine as an adjunctive therapy for paediatric pneumonia compared with conventional Western therapy alone. Specifically, we aim to (1) pool data from all available RCTs to estimate the overall treatment effect on clinical response rates, symptom resolution times, fever duration and hospital stay; (2) compare our findings with those of previous systematic reviews on TCM for paediatric pneumonia, thereby highlighting the unique contribution and limitations of the nebulised route; and (3) provide evidence-based recommendations for both clinical practice and future trial design, with particular emphasis on the urgent need for rigorous placebo-controlled studies. By addressing these objectives, the present study seeks to move beyond general assertions of TCM efficacy and instead offer a methodologically informed, quantitative assessment that can guide clinicians and researchers in this evolving field.

## 2 Methods

### 2.1 Inclusion and exclusion criteria

This systematic review was structured strictly according to the participants, interventions, comparisons, outcomes and study design (PICOS) framework, with specific criteria as follows:

P (participants): The study population included paediatric patients (age ≤ 18 years) with a clinical and/or radiological diagnosis of pneumonia. Studies involving patients with severe underlying diseases (e.g., congenital heart disease, immunodeficiency, chronic lung disease), critical severe pneumonia or severe dysfunction of other organs were excluded.

I (interventions): The intervention for the experimental group was nebulised inhalation of any form of Chinese herbal medicine (compound formula or single herb), used as an adjunct to conventional Western medical therapy. Studies administering Chinese herbs via non-nebulised routes (e.g., intravenous, oral) were excluded.

C (comparisons): The control intervention consisted of identical conventional Western medical therapy as the experimental group, with or without a placebo nebulisation.

O (outcomes): Primary outcomes included the total clinical effective rate and the incidence of adverse reactions. Secondary outcomes comprised the time to improvement of symptoms and signs (fever resolution time, cough resolution time, rales resolution time), time to radiographic improvement and hospital stay duration. Studies that did not report any of the aforementioned relevant outcomes were excluded.

S (study design): Only RCTs were included. Non-randomised studies, observational studies, reviews, case reports, conference abstracts and animal experiments were excluded.

### 2.2 Literature search strategy

A systematic and comprehensive literature search was conducted from the inception of each database to 1 November 2025. The following electronic databases were searched: PubMed, Cochrane Library, Web of Science and Embase (English-language databases); China National Knowledge Infrastructure, Wanfang Data Knowledge Service Platform, VIP Chinese Journal Database and Chinese Biomedical Literature (Chinese-language databases). The search strategy combined subject headings (e.g., Medical Subject Headings terms) and free-text words related to the core concepts: “paediatric pneumonia”, “Chinese herbal medicine”, “nebulised inhalation” or “aerosol inhalation”. The detailed search strategies for each database are provided in Supplementary Table S1. No language restrictions were applied during the search.

### 2.3 Study selection process

The process of study selection was performed independently by two investigators and a third senior investigator. The selection followed a two-phase approach:

Phase 1 (Title/Abstract Screening): After removing duplicate records using EndNote X9 software (Clarivate Plc, Philadelphia, PA, United States), the titles and abstracts of all retrieved records were screened against the predefined inclusion and exclusion criteria. Clearly irrelevant studies (e.g., non-clinical studies, reviews, studies on adult populations) were excluded.

Phase 2 (Full-text Review): The full texts of the remaining potentially eligible articles were obtained and thoroughly assessed for eligibility based on the PICOS criteria detailed.

### 2.4 Data extraction

A pre-designed, standardised data extraction form was used to collect the following information: (1) basic study characteristics (first author, publication year); (2) baseline characteristics of participants (sample size, age, gender); (3) specific details of interventions and comparisons (name of TCM formula, concentration, frequency, duration of treatment, specific conventional therapy regimen); (4) data related to the outcome measures of interest; (5) information required for the risk of bias assessment.

### 2.5 Quality assessment (risk-of-bias assessment)

The methodological quality (risk of bias) of each included RCT was independently assessed by two reviewers using the revised Cochrane risk-of-bias tool (RoB 2) for randomised trials. Any disagreements were resolved through discussion or by consulting a third reviewer. The RoB 2 tool evaluates the risk of bias across five domains: bias arising from the randomisation process, bias due to deviations from intended interventions, bias due to missing outcome data, bias in the measurement of the outcome and bias in the selection of the reported result.

Judgements for each domain were made as “Low risk of bias”, “Some concerns” or “High risk of bias”, based on the detailed criteria and signalling questions provided in the RoB 2 handbook. The overall risk of bias for each study was rigorously determined according to the Cochrane RoB 2 guidance, synthesising the judgements across the aforementioned five domains. The algorithmic logic was as follows: if all domains were judged as “Low risk of bias”, the overall risk was “Low risk”; if one or more domains were judged as raising “Some concerns” but no domain was judged as “High risk of bias”, the overall risk was judged as having “Some concerns”; if any domain was judged as “High risk of bias”, the overall risk of bias for the study was deemed “High risk”, irrespective of judgements in other domains. The risk of bias graph was generated using RevMan 5.4 software.

### 2.6 Statistical analysis

All meta-analyses and heterogeneity tests were performed using Stata 17.0 software. For dichotomous outcomes (e.g., total effective rate), the odds ratio (OR) with a 95% CI was used as the effect measure. For continuous outcomes (e.g., symptom resolution time, hospital stay), the standardised mean difference (SMD) with a 95% CI was employed due to potential differences in measurement scales across studies.

Heterogeneity among studies was assessed using the *I*² statistic. If *I*² ≤ 50% and *p* ≥ 0.1, heterogeneity was considered acceptable, and a fixed-effects model was used for data pooling. If *I*² > 50% and *p* < 0.1, significant heterogeneity was considered present, and a random-effects model was employed.

When a sufficient number of studies were included (typically ≥10), potential publication bias was assessed using Egger's test and visual inspection of funnel plots, performed using Stata 17.0 software.

The conduct and reporting of this review adhered to the Preferred Reporting Items for Systematic Reviews and Meta-Analyses statement (see Supplementary Table S2).

## 3 Results

### 3.1 Literature search and selection results

**Figure 1:**
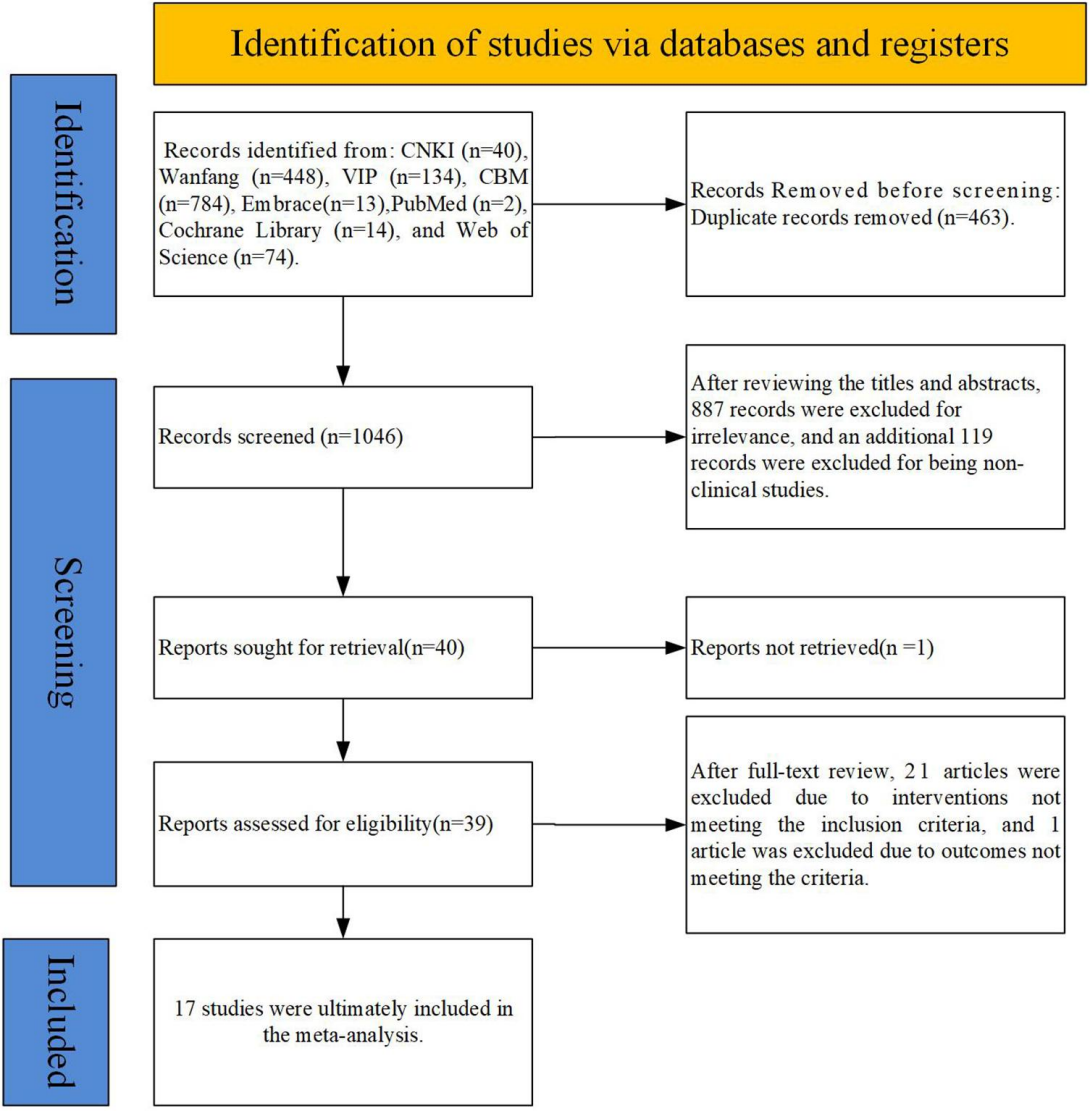
Flow diagram of study selection.

The systematic literature search identified a total of 1,509 records. After removing duplicates and screening titles, abstracts and full texts against the eligibility criteria, 17 RCTs met the inclusion criteria and were included in the meta-analysis (15–31). The detailed flow of the study selection process is presented in Figure 1.

### 3.2 Characteristics of included studies

The basic characteristics of the 17 RCTs included in the final analysis are summarised in Table 1 and Supplementary Table S3. All studies were conducted and published in China, enrolling a total of 1,764 paediatric pneumonia patients, with 915 patients in the treatment groups and 849 in the control groups.

**Table 1:** General characteristics of the included RCTs.

Included study	Study design	Sample size (T/C)	Male	Age (T/C)	Interventions	Outcome indicators
Yan et al. (15)	RCT	60/50	35/30	8 months (mean)/8 months (mean)	T: TCM aerosol inhalation; C: Conventional Western medicine	①
Tuo et al. (16)	RCT	60/60	NR/NR	NR/NR	T: TCM aerosol inhalation; C: Conventional Western medicine	④
Han et al. (17)	RCT	40/40	22/25	<3y:4, 3–7y:30, >7y:6/<3y:3, 3–7y:32, >7y:5	T: TCM aerosol inhalation; C: Conventional Western medicine	①
Wan et al. (18)	RCT	21/21	13/14	22 ± 11.13 months/2.05 ± 1.16 years	T: TCM aerosol inhalation; C: Conventional Western medicine	②③④
Zhi et al. (19)	RCT	36/36	NR/NR	∼3.2 years (mean)/∼3.2 years (mean)	T: TCM aerosol inhalation; C: Conventional Western medicine	①
Zhao et al. (20)	RCT	38/38	22/20	∼4.1 years (mean)/∼6 years (mean)	T: TCM aerosol inhalation; C: Conventional Western medicine	①
Dong et al. (21)	RCT	46/44	28/28	<3y:4, 3–7y:35, >7y:7/<3y:3, 3–7y:36, >7y:5	T: TCM aerosol inhalation; C: Conventional Western medicine	①②③
Bei et al. (22)	RCT	81/51	56/38	4 months-6.5 years (∼1.2 years mean)/4 months-6.5 years (∼1.2 years mean)	T: TCM aerosol inhalation; C: Conventional Western medicine	④
Guan et al. (23)	RCT	50/35	26/20	0.7–12y (∼2.54y mean)/0.5–12y (∼3.58y mean)	T: TCM aerosol inhalation; C: Conventional Western medicine	①
Li et al. (24)	RCT	79/78	NR/NR	2 months-5 years (∼2.5 years mean)/2 months-5 years (∼2.5 years mean)	T: TCM aerosol inhalation; C: Conventional Western medicine	①
Wang et al. (25)	RCT	46/44	NR/NR	3 months-3 years/3 months-3 years	T: TCM aerosol inhalation; C: Conventional Western medicine	③④
Ji et al. (26)	RCT	60/60	33/32	<6m:18, 6–12m:20, 1–2y:12, >2y:10/<6m:20, 6–12m:19, 1–2y:10, >2y:11	T: TCM aerosol inhalation; C: Conventional Western medicine	①
Gui et al. (27)	RCT	68/62	36/36	1 month-3 years (∼1.2 years mean)/1 month-3 years (∼1.2 years mean)	T: TCM aerosol inhalation; C: Conventional Western medicine	①
Sun et al. (28)	RCT	30/30	17/14	3 months-3 years (11.1 ± 5.3 months mean)/3 months-3 years (10.5 ± 4.9 months mean)	T: TCM aerosol inhalation; C: Conventional Western medicine	①②③
Zou et al. (29)	RCT	90/90	55/48	2–11y (3.8 ± 2.0y mean)/1.5–10y (4.2 ± 1.8y mean)	T: TCM aerosol inhalation; C: Conventional Western medicine	①
Yin et al. (30)	RCT	60/60	NR/NR	2 months-2 years (∼1.2 years mean)/2 months-2 years (∼1.2 years mean)	T: TCM aerosol inhalation; C: Conventional Western medicine	①
Tang et al. (31)	RCT	50/50	28/27	<3y:3, 3–7y:28, >7y:19/<3y:2, 3–7y:30, >7y:18	T: TCM aerosol inhalation; C: Conventional Western medicine	①

T, treatment group; C, control group; NR, not reported; RCT, randomized controlled trial; TCM, traditional chinese medicine; y, years; m, months. Outcome Indicators Code: ① (Total Effective Rate); ② (Fever Resolution Time); ③ (Resolution Time of Respiratory Symptoms); ④ (Hospital Stay Duration).

### 3.3 Methodological quality of included studies

**Figure 2:**
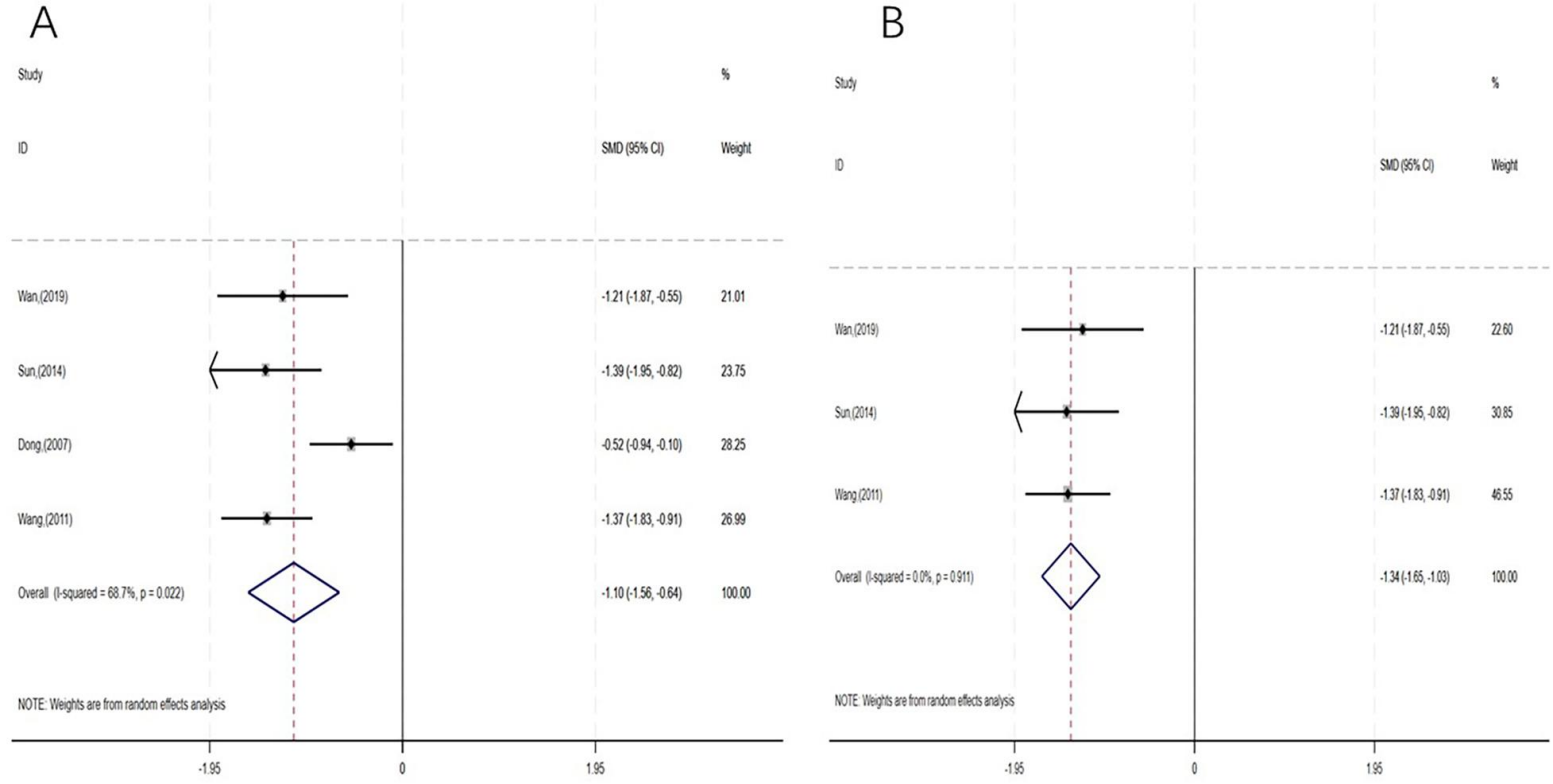
**(A)** Risk of bias summary; **(B)** Risk of bias graph.

The methodological quality of the 17 included studies was assessed using the RoB 2 tool, with the results summarised in Figures 2A,B.

### 3.4 Total effective rate

A total of 13 studies (15, 17, 19–21, 23, 24, 26–31) involving 1,380 patients reported the total clinical effective rate. The test for heterogeneity indicated no heterogeneity among the studies (*I*² = 0.0%, *p* = 0.771); therefore, a fixed-effects model was used for the pooled analysis.

**Figure 3:**
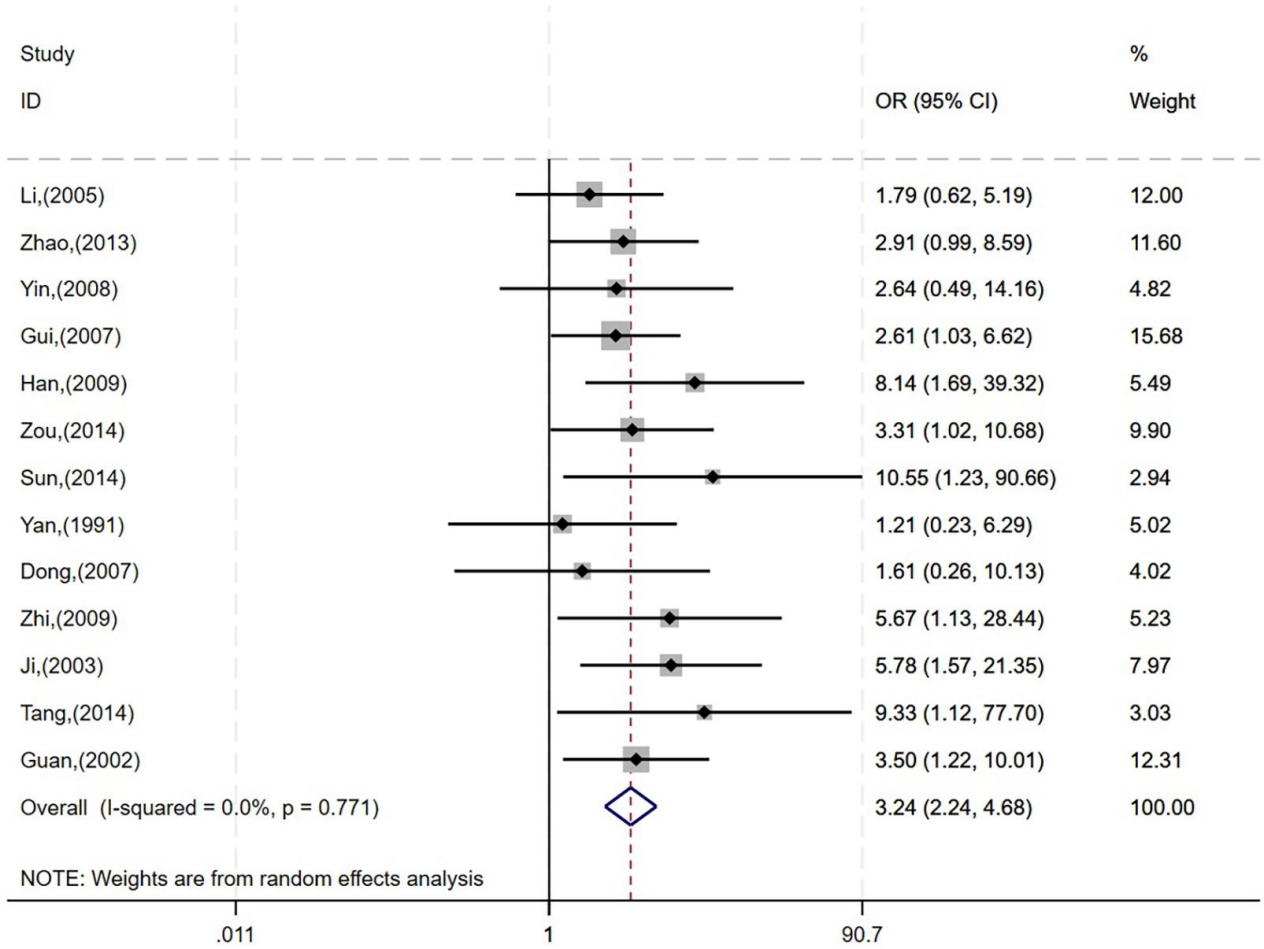
Forest plot of the total effective rate: nebulized Chinese herbal medicine plus conventional therapy vs. conventional therapy alone.

The meta-analysis results (Figure 3) demonstrated that, compared with conventional Western therapy alone, the combination with nebulised Chinese herbal medicine significantly improved the total clinical effective rate, and the difference was statistically significant [pooled OR = 3.24, 95% CI: (2.24, 4.68), *p* < 0.001].

**Figure 4:**
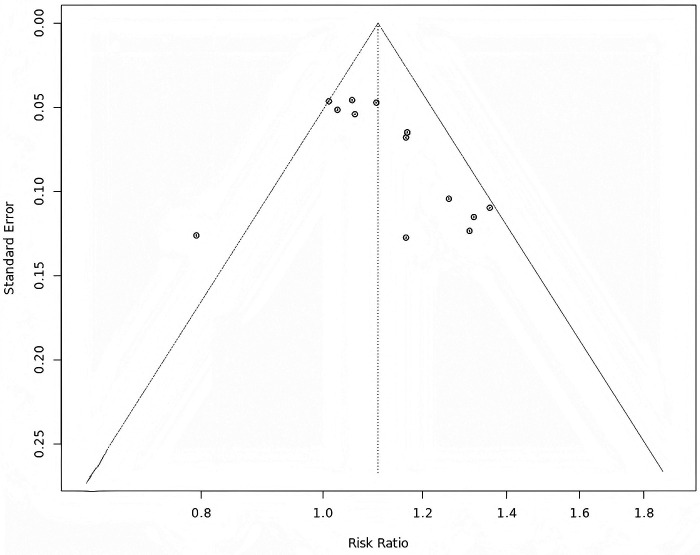
Funnel plot for the comparison of total effective rate.

Publication bias was assessed for the total effective rate. The generated funnel plot appeared approximately symmetrical (Figure 4), suggesting a low likelihood of publication bias.

### 3.5 Resolution time of respiratory symptoms

A total of four studies (18, 21, 25, 28) involving 282 patients reported the resolution time of respiratory symptoms. The test for heterogeneity indicated moderate heterogeneity among the studies (*I*² = 68.7%, *p* = 0.022); therefore, a random-effects model was used for the pooled analysis.

**Figure 5:**
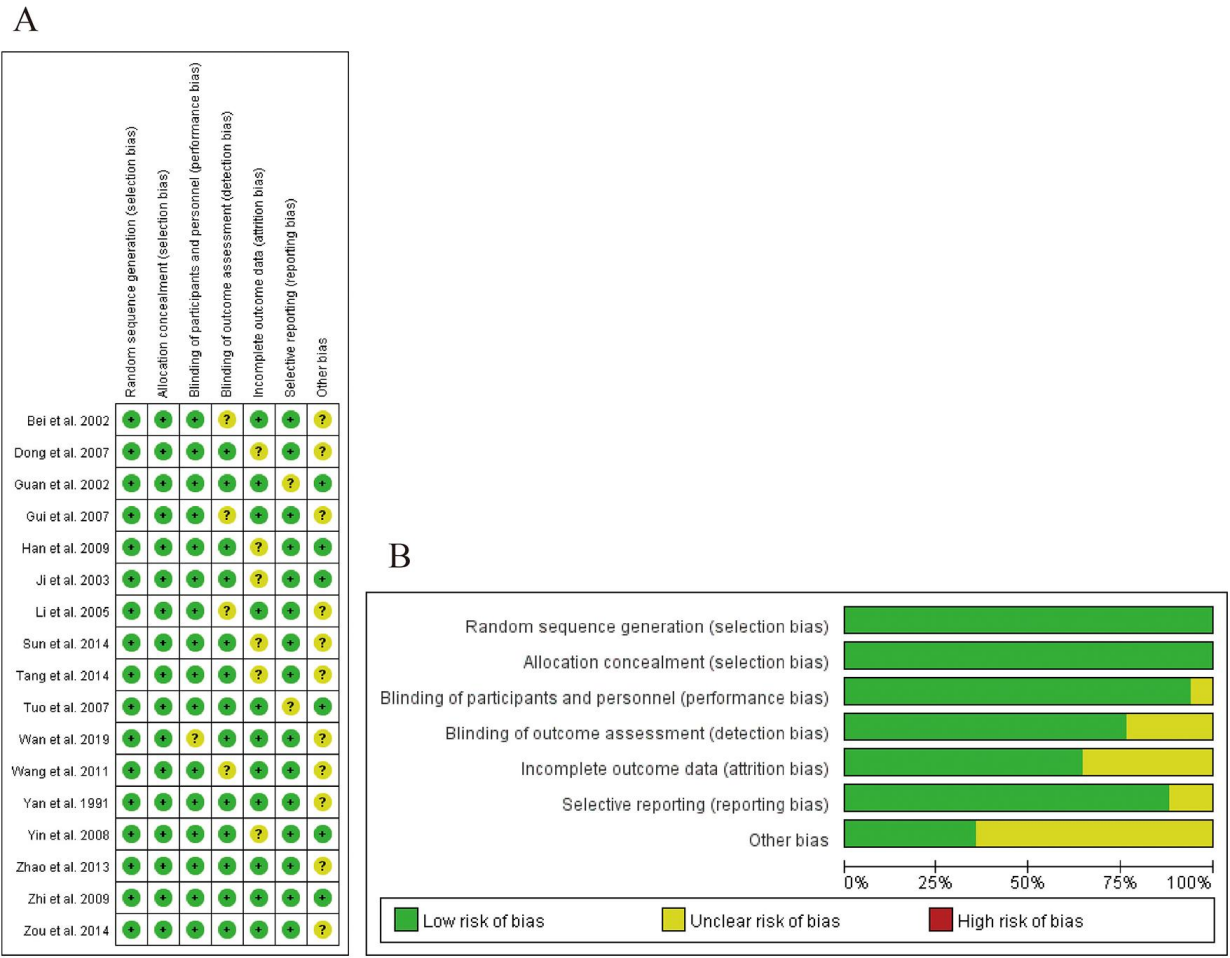
**(A)** Forest plot of the resolution time for respiratory symptoms: nebulized Chinese herbal medicine plus conventional therapy vs. conventional therapy alone; **(B)** Forest plot of the resolution time for respiratory symptoms after excluding the study by Dong et al.

The meta-analysis results (Figure 5A) demonstrated that, compared with the control group, the nebulised Chinese herbal medicine group had a significantly shorter resolution time for respiratory symptoms, and the difference was highly statistically significant [pooled SMD = −1.10, 95% CI: (−1.56, −0.64), *p* < 0.001].

A sensitivity analysis excluding the study by Dong et al. (21), a potential source of heterogeneity, resulted in a more homogeneous set of studies (*I*² = 0.0%) (Figure 5B). This confirms the robustness of the primary finding that adjunctive nebulised Chinese herbal medicine accelerates the resolution of respiratory symptoms.

### 3.6 Fever resolution time

A total of three studies (18, 21, 28) involving 192 patients reported the fever resolution time. The test for heterogeneity indicated high heterogeneity among the studies (*I*² = 70.8%, *p* = 0.033); therefore, a random-effects model was used for the pooled analysis.

**Figure 6:**
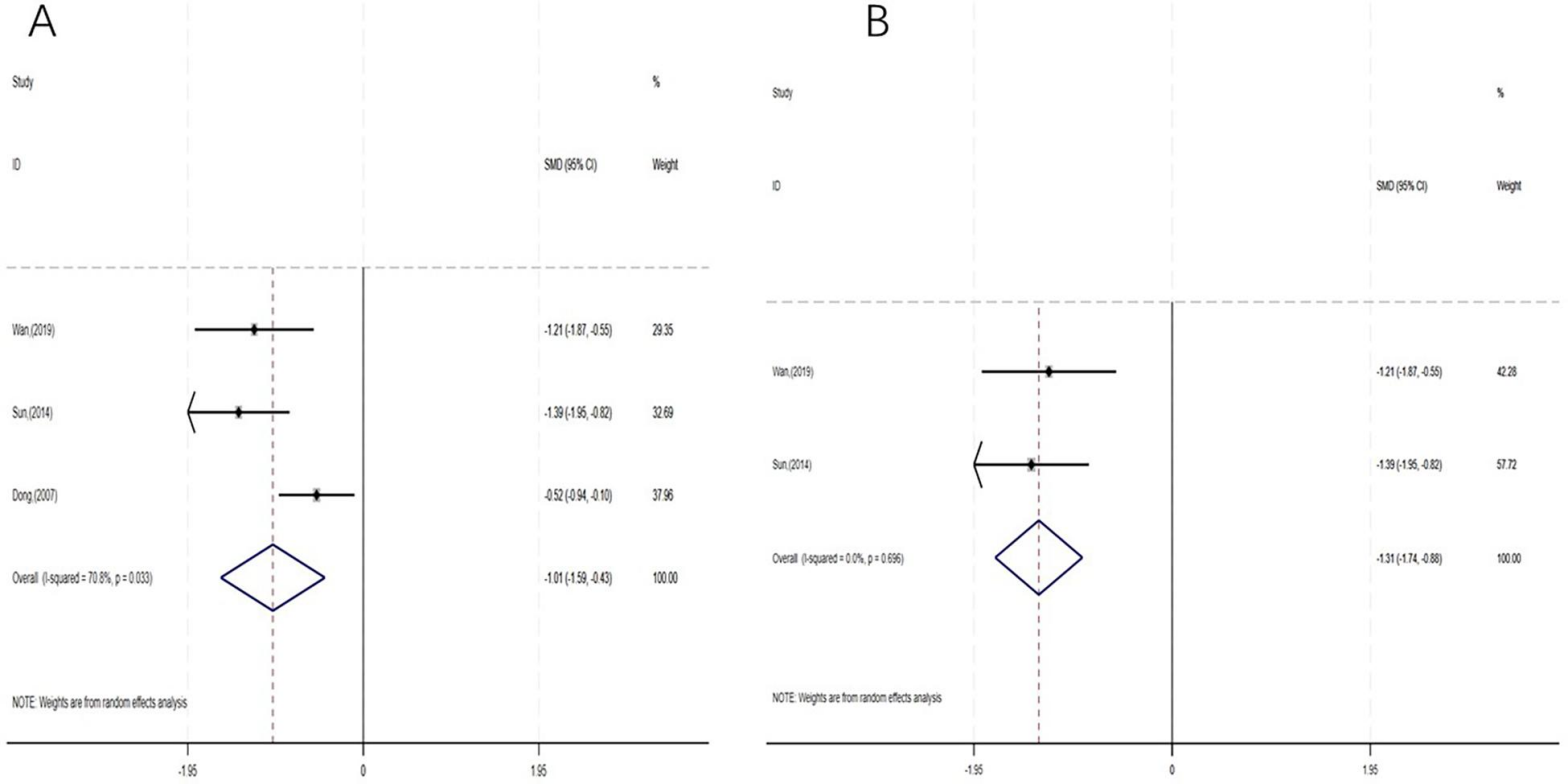
**(A)** Forest plot of the fever resolution time: nebulized Chinese herbal medicine plus conventional therapy vs. conventional therapy alone; **(B)** Forest plot of fever resolution time after excluding the study by Dong et al.

The meta-analysis results (Figure 6A) demonstrated that, compared with the control group, the nebulised Chinese herbal medicine group had a significantly shorter fever resolution time, and the difference was highly statistically significant [pooled SMD = −1.01, 95% CI: (−1.59, −0.43), *p* = 0.0006].

A sensitivity analysis, performed by removing the study by Dong et al. (21), which contributed substantially to the heterogeneity, yielded a consistent but more precise effect estimate (SMD = −1.31) with no residual heterogeneity (*I*² = 0.0%) (Figure 6B).

### 3.7 Hospital stay duration

A total of four studies (16, 18, 22, 25) involving 384 patients reported the hospital stay duration. The test for heterogeneity indicated moderate heterogeneity among the studies (*I*² = 52.7%, *p* = 0.096); therefore, a random-effects model was used for the pooled analysis.

**Figure 7:**
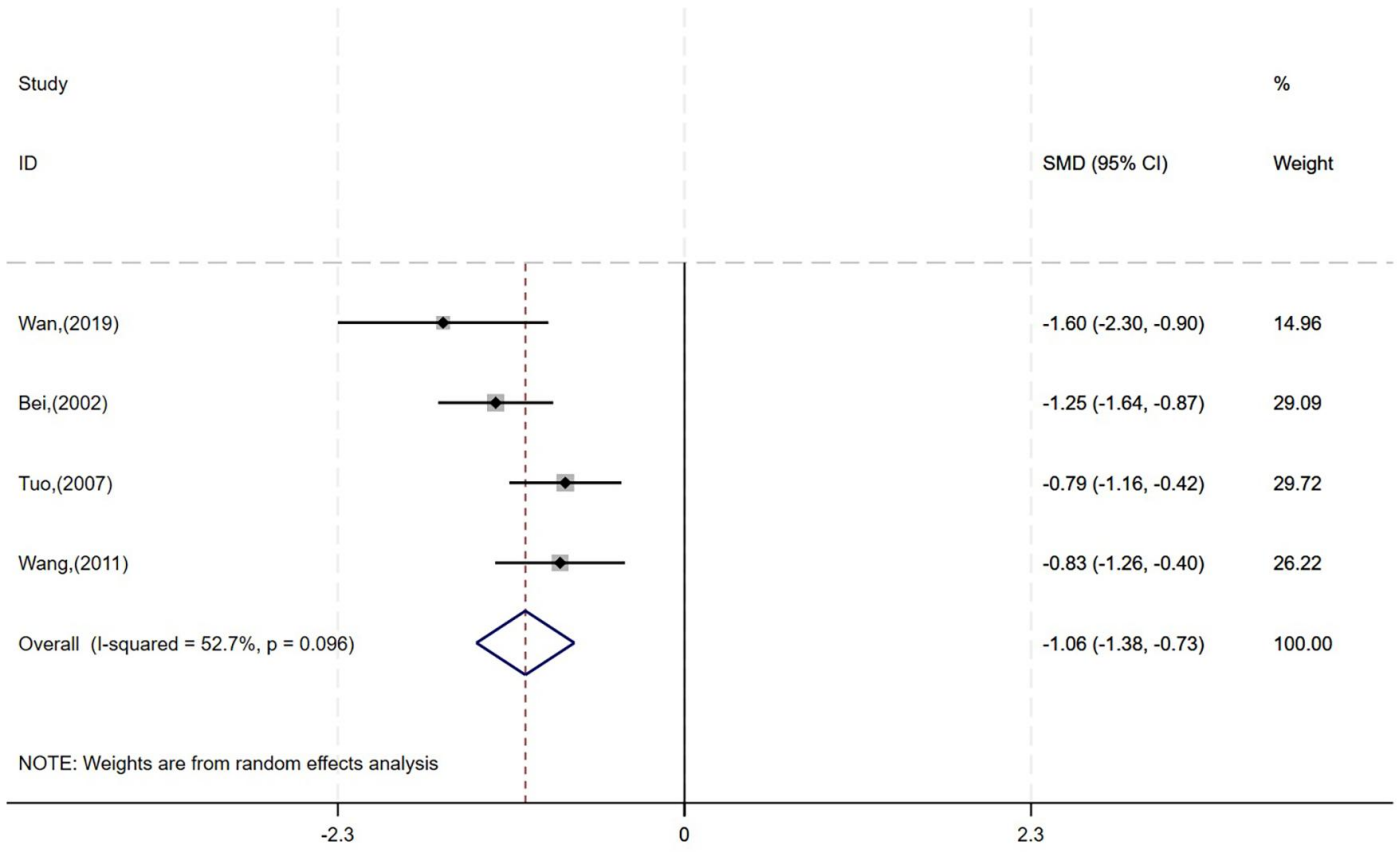
Forest plot of the hospital stay duration: nebulized Chinese herbal medicine plus conventional therapy vs. conventional therapy alone.

The meta-analysis results (Figure 7) demonstrated that, compared with the control group, the nebulised Chinese herbal medicine group had a significantly shorter hospital stay, and the difference was highly statistically significant [pooled SMD = −1.06, 95% CI: (−1.38, −0.73), *p* < 0.001].

This result indicates that adjunctive therapy with nebulised Chinese herbal medicine can effectively reduce the hospitalisation course for paediatric patients with pneumonia. The pooled effect size (SMD of −1.06) represents a large effect size according to Cohen's criteria, demonstrating important clinical significance for this intervention in reducing hospital stay.

## 4 Discussion

This systematic review and meta-analysis of 17 RCTs and 1,764 paediatric patients provides a robust quantitative synthesis specifically focused on the adjunctive use of nebulised Chinese herbal medicine for paediatric pneumonia. The principal findings indicate that, compared with conventional Western therapy alone, the addition of nebulised TCM significantly improves the total clinical effectiveness rate and accelerates the resolution of key symptoms, including respiratory manifestations and fever, ultimately leading to a shorter hospital stay.

### 4.1 Comparison with previous studies

Our findings align with previous systematic reviews on TCM for respiratory infections. For instance, a systematic review by Guo et al. (7) concluded that TCM as an adjunctive therapy could improve clinical efficacy and shorten fever time in children with CAP, which is consistent with our findings. However, our analysis is the first to focus specifically on the nebulised inhalation route of administration within the paediatric pneumonia population, providing stronger evidence through quantitative pooled effect estimates than previous narrative reviews. This study confirms that nebulised inhalation, as a localised and targeted therapy, directly delivers active herbal ingredients to the site of disease, which may be key to its significant therapeutic effects (32, 33).

### 4.2 Possible explanations and mechanisms

The effectiveness of nebulised TCM for paediatric pneumonia can be explained through both modern pharmacology and TCM theory. From a modern scientific perspective, nebulisation enables the direct delivery of active TCM components (e.g., flavonoids, alkaloids, volatile oils) to the respiratory tract mucosa and alveoli, achieving high local drug concentrations. This allows for the full exertion of pharmacological effects, such as anti-inflammatory, antiviral, antibacterial, sputum-diluting and airway mucosa-repairing actions (34). Many Chinese herbs have been demonstrated to possess immunomodulatory functions, potentially mitigating the excessive inflammatory response associated with pneumonia (35). From the TCM theoretical perspective, pneumonia falls under the categories of “cough” and “pneumonia with dyspnoeic cough”, primarily attributed to pathogenic factors invading the lung defence system and resulting in lung qi stagnation. Inhaling acrid–cool or acrid–warm dispersing herbs directly promotes the diffusion of lung qi and expels pathogens, aligning with the therapeutic principle of “treating the upper energiser like a feather, using light and ascending agents”, thereby facilitating a rapid onset of action (36, 37).

However, a critical methodological limitation must be acknowledged: among the 17 included RCTs, none employed placebo nebulisation (e.g., isotonic or hypertonic saline) in the control group. The comparator was conventional Western therapy alone, without any nebulised vehicle. This design introduces potential confounding, as nebulised isotonic or hypertonic solutions themselves are known to improve mucociliary clearance, hydrate airway surfaces and facilitate sputum expectoration—effects that could independently contribute to symptom relief and shorter hospital stays (13, 14). Notably, recent meta-analyses have demonstrated that even nebulised 0.9% normal saline—often regarded as an “inert placebo”—exhibits measurable therapeutic benefits in paediatric respiratory diseases. For instance, Feng et al. (2024) showed that nebulised 3% hypertonic saline significantly reduced the length of hospital stay, decreased the rate of hospitalisation, improved clinical severity scores and enhanced respiratory distress compared with 0.9% normal saline in infants with acute bronchiolitis (10). Furthermore, a systematic review by House et al. (11) found that nebulised normal saline produced significant improvements in respiratory scores within 60 min of treatment, with an SMD of −0.9 [95% CI: (−1.2, −0.6)] compared with other placebo controls (11). Beyond individual trials, national expert consensus has recognised the clinically relevant impact of inhaled muco-active agents on outcomes of various lower respiratory tract infections in children. Singh et al. (12), representing an Indian expert group, recommended inhaled muco-active drugs (including hypertonic saline) as adjunctive therapy for paediatric respiratory diseases, such as bronchiolitis, pneumonia and bronchiectasis, based on evidence of improved symptom clearance and reduced hospitalisation duration (12). This further underscores that nebulisation *per se*—regardless of the specific agent—confers therapeutic benefits through mechanical and hydrating effects on airway secretions.

Collectively, these findings indicate that nebulised saline (whether isotonic or hypertonic) exerts non-specific muco-active effects beyond simple humidification. Therefore, the observed benefits in our meta-analysis cannot be unequivocally attributed solely to the herbal constituents; a non-specific effect of the nebulisation procedure itself may account for part of the treatment effect. Future trials should include a placebo arm (e.g., nebulised saline with matching appearance and taste, if feasible) to isolate the specific pharmacological contribution of Chinese herbal ingredients. Without such controls, the current evidence, although promising, remains susceptible to overestimation of the true treatment effect.

### 4.3 Limitations of the review

Several limitations of this review should be acknowledged. First, and most importantly, a critical confounding factor exists in all included studies: none employed placebo nebulisation (e.g., isotonic or hypertonic saline) in the control group. The comparator was conventional Western therapy alone, without any nebulised vehicle. This design precludes the ability to distinguish between the specific pharmacological effects of Chinese herbal ingredients and the well-documented non-specific therapeutic effects of nebulisation itself. Accumulating evidence demonstrates that even nebulised 0.9% normal saline has important muco-active properties, including enhanced mucociliary clearance, airway hydration and sputum expectoration, which can independently improve respiratory symptoms and shorten hospital stays (38–41). National expert consensus has further recognised that inhaled muco-active agents (including nebulised saline) produce clinically relevant improvements in paediatric lower respiratory tract infections (12). Consequently, the observed benefits in our meta-analysis may be partially or substantially attributable to the nebulisation procedure rather than the herbal components *per se*. Without a placebo arm, the true specific treatment effect of Chinese herbal medicine remains uncertain, and the current evidence is susceptible to overestimation. Second, although all included studies reported positive outcomes, the possibility of publication bias cannot be entirely excluded, although the funnel plot for the primary outcome was symmetrical. The exclusively Chinese origin of the studies may affect the generalisability of findings to other ethnic populations and healthcare systems. Third, clinical and methodological heterogeneity was present, particularly for continuous outcomes such as symptom duration. Variability in TCM formulas, pneumonia aetiologies (viral, bacterial, mycoplasmal) and patient age ranges likely contributed. Fourth, the quality of reporting in many primary studies was suboptimal, with insufficient details on randomisation procedures, allocation concealment and blinding of outcome assessors, which may introduce additional bias.

### 4.4 Implications for practice and research

Based on the current evidence, nebulised TCM can be considered a potentially effective adjunctive option to conventional Western therapy for paediatric pneumonia, particularly in clinical scenarios aiming for rapid symptom relief and shortened hospitalisation (7, 32, 38–41). When applying this therapy, clinicians should pay attention to syndrome differentiation in formula selection and closely monitor potential adverse reactions.

Future research should focus on conducting larger, multicentre RCTs with more rigorous designs, utilising standardised TCM preparations and providing detailed reporting on allocation concealment and blinding procedures. Crucially, future trials must include a placebo arm with nebulised saline (isotonic or hypertonic, as appropriate) to control for the non-specific muco-active effects of nebulisation itself, thereby enabling accurate estimation of the specific therapeutic contribution of herbal ingredients. Beyond improved trial design, advances in drug delivery technology offer promising avenues for enhancing the efficacy of inhaled herbal medicine. Nanoparticle-based inhalation therapy represents a cutting-edge approach that enables direct administration of therapeutics to the lung, maintaining high topical drug concentrations while minimising systemic exposure (42, 43). This strategy has shown potential in various pulmonary diseases by improving drug stability, sustained release and targeted delivery to specific cell populations within the respiratory tract. For Chinese herbal medicine, encapsulation of active phytochemicals (e.g., flavonoids, alkaloids, volatile oils) into biodegradable nanoparticles could address current limitations, such as the rapid clearance, variable absorption and irritation potential of crude herbal extracts.

Recent developments in targeted inhalation herbal therapy have demonstrated remarkable success beyond infectious diseases. For instance, Gaber et al. (44) developed inhalable herbal nanotherapeutics that achieved efficient anti-tumour effects against lung carcinoma, with preferential pulmonary deposition and low off-target biodistribution (44). This strategy, leveraging the natural bioactivity of herbal compounds combined with nanoparticle-based targeted delivery, significantly enhanced local drug accumulation at disease sites while reducing systemic toxicity. Such innovative approaches could be adapted for paediatric pneumonia by designing pathogen-targeted or inflammation-targeted herbal nanoparticles, potentially improving therapeutic index and reducing dosing frequency. Future research should explore the development of herbal-derived nanoparticle formulations for nebulised delivery, along with rigorous preclinical and clinical evaluations of their safety and efficacy in paediatric pneumonia, drawing inspiration from these successful anticancer applications. Studies should further define the optimal formula composition, dosage and treatment duration and explore its efficacy against pneumonia caused by specific pathogens (e.g., RSV, *Mycoplasma pneumoniae*), thereby providing higher-level evidence for clinical practice.

## Data Availability

The original contributions presented in the study are included in the article/Supplementary Material, further inquiries can be directed to the corresponding author.
