# Supplementary material for: Nebulised Chinese herbal medicine for paediatric pneumonia: a meta-analysis
Source: Front Pediatr. 2026 Jul 3;14:1777233. doi: 10.3389/fped.2026.1777233 (PMC13375793; doi:10.3389/fped.2026.1777233)
Supplement: Supplementary file 1 [file Table1.docx]

| Supplementary Table 1 Search Strategy |
| --- |
| CNKI |
| SU=infantile pneumonia OR pediatric pneumonia OR infant and young child pneumonia) AND (SU=traditional Chinese medicine nebulization inhalation OR traditional Chinese medicine nebulization OR Chinese herbal medicine nebulization OR traditional Chinese medicine (TCM) nebulization) AND (SU=randomized controlled trial OR controlled trial OR controlled study OR randomized trial OR randomized study OR clinical study OR clinical trial OR clinical control OR cluster randomized OR cluster controlled OR parallel controlled OR crossover trial OR crossover intervention OR crossover controlled OR open-label OR single-blind OR double-blind OR triple-blind |
| Pubmed |
| ("Pneumonia"[Mesh] OR "pneumonia" OR "bronchopneumonia")  AND ("child"[Mesh] OR "infant"[Mesh] OR "pediatric" OR "children")  AND ("Drugs, Chinese Herbal"[Mesh] OR "Medicine, Chinese Traditional"[Mesh] OR "herbal medicine" OR "Chinese medicine")  AND ("Nebulizers and Vaporizers"[Mesh] OR "inhalation" OR "nebulization" OR "atomization")  AND ("Randomized Controlled Trial"[Publication Type] OR "randomized" OR "RCT")  Filters: Publication date from 1900/01/01 to 2025/11/1; Journal Article; English |
| Embase |
| 'pneumonia'/exp OR 'childhood pneumonia' OR 'pediatric pneumonia'  AND 'child'/exp OR 'infant'/exp  AND 'traditional chinese medicine'/exp OR 'chinese herbal medicine' OR 'herbal medicine'  AND 'nebulization'/exp OR 'inhalation' OR 'aerosol therapy'  AND 'randomized controlled trial'/exp OR 'randomized'  AND [english]/lim AND [1-1-1900]/sd NOT [1-11-2025]/sd  AND [embase]/lim NOT [medline]/lim |
| Web of Science |
| TS=("pneumonia" OR "bronchopneumonia") AND TS=("child*" OR "pediatric" OR "infant*")  AND TS=("Chinese herbal*" OR "traditional Chinese medicine" OR "herbal medicine")  AND TS=("nebuliz*" OR "inhal*" OR "atomiz*")  AND TS=("randomized" OR "RCT" OR "randomly allocated")  AND LANGUAGE: (English) AND DOCUMENT TYPES: (Article)  Time span: 1900-2025 |
| Wanfang Database |
| Topic: (Infantile Pneumonia OR Childhood Pneumonia OR Infant Pneumonia OR Bronchopneumonia) AND Topic: (Traditional Chinese Medicine Nebulization OR Chinese Herbal Medicine Nebulization OR Nebulized Inhalation) AND Topic: (Randomized OR RCT OR Randomized Controlled) |
| VIP Database |
| Topic=((Infantile Pneumonia + Childhood Pneumonia + Bronchopneumonia + Mycoplasma Pneumonia) * (Traditional Chinese Medicine Nebulization + Chinese Herbal Medicine Nebulization + Nebulized Inhalation + Traditional Chinese Medicine Nebulized Inhalation) * (Randomized + RCT + Randomized Controlled + Controlled Trial)) Publication Year <= 2025 |
| Chinese Biomedical Literature Database，CBM |
| (("Pneumonia, Child"[MH] OR "Child Pneumonia"[TIAB] OR "Pediatric Pneumonia"[TIAB] OR "Infant Pneumonia"[TIAB] OR "Pneumonia in children"[TIAB] OR "Pediatric Pulmonary Infection"[TIAB] OR "Child Pneumonia"[KW] OR "Pediatric Pneumonia"[KW]) AND ("Drugs, Chinese Herbal"[MH] OR "Chinese Herbal Medicine"[TIAB] OR "Chinese Herbal Drugs"[TIAB] OR "Traditional Chinese Medicine"[TIAB] OR "Herbal Medicine"[TIAB] OR "Chinese Herb*"[TIAB] OR "Chinese Herbal Medicine"[KW] OR "Chinese Herbal Drugs"[KW] OR "Traditional Chinese Medicine"[KW]) AND ("Nebulizers and Vaporizers"[MH] OR "Inhalation"[MH] OR "Nebuliz*"[TIAB] OR "Inhal*"[TIAB] OR "Aerosol*"[TIAB] OR "Atomiz*"[TIAB] OR "Nebuliz*"[KW] OR "Inhal*"[KW] OR "Aerosol*"[KW])) AND (PY <= 2025) |
| Cochrane Library |
| #1 MeSH descriptor: [Pneumonia] this term only  #2 MeSH descriptor: [Child] this term only  #3 (pneumon or bronchopneumon or "lung inflammation"):ti,ab,kw  #4 (child or pediatric or paediatric or infant or toddler or baby or babies or boy or girl or schoolchild or preschool or kindergarten or adolescent or teen or juvenil or minor or underag* or under-ag or youth):ti,ab,kw  #5 #1 AND #2  #6 #3 AND #4  #7 #5 OR #6  #8 MeSH descriptor: [Drugs, Chinese Herbal] this term only  #9 ("Chinese herb" or "Chinese medicine" or "Chinese drug" or "traditional Chinese medicine" or TCM or "herbal medicine" or "herbal drug" or phytotherapy or "plant extract" or "medicinal plant"):ti,ab,kw  #10 #8 OR #9  #11 MeSH descriptor: [Nebulizers and Vaporizers] this term only  #12 MeSH descriptor: [Inhalation] this term only  #13 (nebuliz or nebuliser or nebulizer or atomiz or inhal or aerosol or vaporiz or vapour or mist or spray or sprays or sprayed):ti,ab,kw  #14 #11 OR #12 OR #13  #15 #7 AND #10 AND #14 |
