# Supplementary material for: Nebulised Chinese herbal medicine for paediatric pneumonia: a meta-analysis
Source: Front Pediatr. 2026 Jul 3;14:1777233. doi: 10.3389/fped.2026.1777233 (PMC13375793; doi:10.3389/fped.2026.1777233)
Supplement: Supplementary file 3 [file Table3.docx]

**Supplementary Table 3 Detailed Information of Nebulized Chinese Herbal Medicine Interventions in Included Studies**

| Study ID | TCM Formula Name | Composition (Herbal Ingredients) | Dosage per Nebulization | Frequency | Duration (Days) |
| --- | --- | --- | --- | --- | --- |
| Yan et al. 1991 [15] | Formula 1, 2, 3 (Individualized by syndrome) | **Formula 1**: *Ephedra sinica* Stapf (*Mahuang*), *Asarum sieboldii* Miq. (*Xixin*), *Pinellia ternata* (Thunb.) Breitenb. (*Banxia*), *Tussilago farfara* L. (*Kuandonghua*), *Aristolochia debilis* Siebold & Zucc. (*Madouling*), *Forsythia suspensa* (Thunb.) Vahl (*Lianqiao*), *Lonicera japonica* Thunb. (*Yinhua*), *Scutellaria baicalensis* Georgi (*Huangqin*) **Formula 2**: *Sparganium stoloniferum* (Buch.-Ham. ex Graebn.) Buch.-Ham. ex Juz. (*Sanleng*), *Curcuma zedoaria* (Christm.) Roscoe (*Ezhu*), *Pinellia ternata* (Thunb.) Breitenb. (*Banxia*), *Descurainia sophia* (L.) Webb ex Prantl (*Liaozi*), *Scutellaria baicalensis* Georgi (*Huangqin*), *Lonicera japonica* Thunb. (*Yinhua*), *Aristolochia debilis* Siebold & Zucc. (*Madouling*) **Formula 3**: *Glehnia littoralis* F.Schmidt ex Miq. (*Shashen*), *Ophiopogon japonicus* (Thunb.) Ker Gawl. (*Maidong*), *Scrophularia ningpoensis* Hemsl. (*Yuanshen*), *Forsythia suspensa* (Thunb.) Vahl (*Lianqiao*), *Polygala tenuifolia* Willd. (*Yuanzhi*), *Tussilago farfara* L. (*Kuandonghua*), *Pseudostellaria heterophylla* (Miq.) Pax (*Taizishen*) | 5 mL extract + 15 mL saline | 3-4 times/day | 7-10 |
| Tuo et al. 2007 [16] | Maxing Shigan Tang Jiawei | *Ephedra sinica* Stapf (*Mahuang*), *Prunus armeniaca* L. (*Xingren*), *Gypsum fibrosum* (*Shigao*; mineral), *Glycyrrhiza uralensis* Fisch. ex DC. (*Gancao*), *Citrus reticulata* Blanco (*Chenpi*), *Houttuynia cordata* Thunb. (*Yuxingcao*), *Fritillaria thunbergii* Miq. (*Zhebeimu*), *Meretrix meretrix* shell (*Haifen*; animal) | Not specified (decoction concentrated to 100 mL total) | 3-4 times/day | Until symptoms resolve |
| Han et al. 2009 [17] | Self-made formula | *Ephedra sinica* Stapf (*Sheng Mahuang*), *Prunus armeniaca* L. (*Xingren*), *Platycodon grandiflorus* (Jacq.) A.DC. (*Jiegeng*), *Glycyrrhiza uralensis* Fisch. ex DC. (*Gancao*), *Lonicera japonica* Thunb. (*Yinhua*). Additions: *Glehnia littoralis* F.Schmidt ex Miq. (*Shashen*), *Ophiopogon japonicus* (Thunb.) Ker Gawl. (*Maidong*), *Fritillaria cirrhosa* D. Don (*Chuanbeimu*) for dry cough; *Astragalus membranaceus* (Fisch.) Bunge (*Sheng Huangqi*), *Atractylodes macrocephala* Koidz. (*Baizhu*), *Triticum aestivum* L. (*Fuxiaomai*) for fatigue/sweating | 20 mL extract + 15 mL saline | 2 times/day | 7 |
| Wan et al. 2019 [18] | Self-made formula | *Prunus armeniaca* L. (*Xingren*), *Aster tataricus* L. f. (*Ziwan*), *Tussilago farfara* L. (*Kuandonghua*), *Belamcanda chinensis* (L.) Redouté (*Shegan*), *Paeonia lactiflora* Pall. (*Baishao*) | 3-10 mL concentrated decoction | 3 times/day | 7 |
| Zhi et al. 2009 [19] | Self-made formula (two stages) | Early stage: *Ephedra sinica* Stapf (*Sheng Mahuang*), *Prunus armeniaca* L. (*Xingren*), *Citrus reticulata* Blanco (*Chenpi*), *Pinellia ternata* (Thunb.) Breitenb. (*Banxia*), *Platycodon grandiflorus* (Jacq.) A.DC. (*Jiegeng*), *Peucedanum praeruptorum* Dunn (*Qianhu*), *Glycyrrhiza uralensis* Fisch. ex DC. (*Gancao*)  Late stage: *Glehnia littoralis* F.Schmidt ex Miq. (*Shashen*), *Ophiopogon japonicus* (Thunb.) Ker Gawl. (*Maidong*), *Polygonatum odoratum* (Mill.) Druce (*Yuzhu*) | 20 mL extract + 15 mL saline | 2 times/day | 7 |
| Zhao et al. 2013 [20] | Qingxuan Heji | *Ephedra sinica* Stapf (*Zhi Mahuang*), *Prunus armeniaca* L. (*Xingren*), *Gypsum fibrosum* (*Sheng Shigao*; mineral), *Scutellaria baicalensis* Georgi (*Huangqin*), *Schizonepeta tenuifolia* (Benth.) Briq. (*Jingjiesui*), *Isatis indigotica* Fortune (*Banlangen*), *Houttuynia cordata* Thunb. (*Yuxingcao*), *Lonicera japonica* Thunb. (*Yinhua*), *Forsythia suspensa* (Thunb.) Vahl (*Lianqiao*), *Anemarrhena asphodeloides* Bunge (*Zhimu*), *Bupleurum chinense* DC. (*Chaihu*), *Glycyrrhiza uralensis* Fisch. ex DC. (*Gancao*) | 30-80 mL decoction | 3 times/day | 7 |
| Dong et al. 2007 [21] | Self-made formula | *Lonicera japonica* Thunb. (*Yinhua*), *Scutellaria baicalensis* Georgi (*Huangqin*), *Forsythia suspensa* (Thunb.) Vahl (*Lianqiao*), *Tussilago farfara* L. (*Kuandonghua*), *Pinellia ternata* (Thunb.) Breitenb. (*Banxia*), *Trichosanthes kirilowii* Maxim. (*Gualou*), *Morus alba* L. (*Sangbaipi*), *Perilla frutescens* (L.) Britton (*Zisuzi*), *Peucedanum praeruptorum* Dunn (*Qianhu*) | 10 mL extract + 10 mL saline | 2 times/day | 7–10 |
| Bei et al. 2002 [22] | Yujin Injection | *Curcuma aromatica* Salisb. (*Yujin*) injection | 1-2 mL + 10 mL saline | 2 times/day | Not specified |
| Guan et al. 2002 [23] | Modified Yinqiao San + Maxing Shigan Tang / Modified Wuhu Tang + Tingli Dazao Xiefei Tang | Wind-heat type: *Lonicera japonica* Thunb. (*Yinhua*), *Forsythia suspensa* (Thunb.) Vahl (*Lianqiao*), *Ephedra sinica* Stapf (*Zhi Mahuang*), *Prunus armeniaca* L. (*Xingren*), *Glycyrrhiza uralensis* Fisch. ex DC. (*Sheng Gancao*), *Gypsum fibrosum* (*Sheng Shigao*; mineral), *Platycodon grandiflorus* (Jacq.) A.DC. (*Jiegeng*), *Arctium lappa* L. (*Niubangzi*), *Morus alba* L. (*Sangbaipi*), *Fritillaria thunbergii* Miq. (*Zhebei*) Phlegm-heat type: *Ephedra sinica* Stapf (*Zhi Mahuang*), *Prunus armeniaca* L. (*Chao Xingren*), *Gypsum fibrosum* (*Sheng Shigao*; mineral), *Glycyrrhiza uralensis* Fisch. ex DC. (*Gancao*), *Descurainia sophia* (L.) Webb ex Prantl (*Tinglizi*), *Scutellaria baicalensis* Georgi (*Huangqin*), *Paeonia veitchii* Lynch (*Chishao*), *Houttuynia cordata* Thunb. (*Yuxingcao*), *Trichosanthes kirilowii* Maxim. (*Quangualou*), *Citrus reticulata* Blanco (*Chenpi*), *Pinellia ternata* (Thunb.) Breitenb. (*Banxia*), *Salvia miltiorrhiza* Bunge (*Danshen*), *Prunus persica* (L.) Batsch (*Taoren*) | 20 mL (1 dose) | 2 times/day | 5–7 |
| Li et al. 2005 [24] | Huatan Pingchuan Tang | *Ephedra sinica* Stapf (*Zhi Mahuang*), *Prunus armeniaca* L. (*Xingren*), *Pheretima aspergillum* (*Dilong*; animal), *Bombyx batryticatus* (*Jiangcan*; animal), *Carthamus tinctorius* L. (*Honghua*), *Asarum sieboldii* Miq. (*Xixin*), *Tussilago farfara* L. (*Kuandonghua*), *Belamcanda chinensis* (L.) Redouté (*Shegan*), *Ginkgo biloba* L. (*Baiguo*), *Descurainia sophia* (L.) Webb ex Prantl (*Tinglizi*) | 20 mL injection | 2 times/day | 7 |
| Wang et al. 2011 [25] | Self-made formula | *Ephedra sinica* Stapf (*Zhi Mahuang*), *Prunus armeniaca* L. (*Xingren*), *Perilla frutescens* (L.) Britton (*Sulizi*), *Descurainia sophia* (L.) Webb ex Prantl (*Tinglizi*), *Raphanus sativus* L. (*Laifuzi*), *Pinellia ternata* (Thunb.) Breitenb. (*Banxia*), *Poria cocos* (Schwein.) F.A. Wolf (*Fuling*), *Citrus reticulata* Blanco (*Chenpi*), *Plantago asiatica* L. (*Cheqianzi*), *Zingiber officinale* Roscoe (*Ganjiang*), *Glycyrrhiza uralensis* Fisch. ex DC. (*Gancao*) | 100 mL decoction (total) | 2 times/day | Until symptoms resolve |
| Ji et al. 2003 [26] | Self-made formula | *Ephedra sinica* Stapf (*Mahuang*), *Schizonepeta tenuifolia* (Benth.) Briq. (*Jingjie*), *Angelica sinensis* (Oliv.) Diels (*Danggui*), *Scutellaria baicalensis* Georgi (*Huangqin*), *Phellodendron amurense* Rupr. (*Huangbai*), *Platycodon grandiflorus* (Jacq.) A.DC. (*Jiegeng*), *Glycyrrhiza uralensis* Fisch. ex DC. (*Gancao*), *Pinellia ternata* (Thunb.) Breitenb. (*Banxia*), *Forsythia suspensa* (Thunb.) Vahl (*Lianqiao*), *Houttuynia cordata* Thunb. (*Yuxingcao*), *Lonicera japonica* Thunb. (*Yinhua*), *Fritillaria cirrhosa* D. Don (*Chuanbei*), *Asarum sieboldii* Miq. (*Xixin*) | 10 mL extract + 10 mL saline | 2 times/day | 7–10 |
| Gui et al. 2007 [27] | Yuxingcao Injection | *Houttuynia cordata* Thunb. injection | 20 mL + 20 mL distilled water | 2 times/day | Not specified |
| Sun et al. 2014 [28] | Qingfei Tang | *Ophiopogon japonicus* (Thunb.) Ker Gawl. (*Maidong*), *Asparagus cochinchinensis* (Lour.) Merr. (*Tiandong*), *Anemarrhena asphodeloides* Bunge (*Zhimu*), *Fritillaria thunbergii* Miq. (*Beimu*), *Glycyrrhiza uralensis* Fisch. ex DC. (*Gancao*), *Citrus reticulata* Blanco (*Juhong*), *Scutellaria baicalensis* Georgi (*Huangqin*), *Morus alba* L. (*Sangbaipi*) | 1 dose decoction (divided for nebulization) | 2 times/day | 7 |
| Zou et al. 2014 [29] | Self-made Formula II | *Ephedra sinica* Stapf (*Mahuang*), *Lonicera japonica* Thunb. (*Yinhua*), *Prunus armeniaca* L. (*Xingren*), *Houttuynia cordata* Thunb. (*Yuxingcao*), *Pheretima aspergillum* (*Dilong*; animal), *Glycyrrhiza uralensis* Fisch. ex DC. (*Gancao*) | Not specified (decoction, filtered) | Not specified | 7 |
| Yin et al. 2008 [30] | Asarone Injection (Xixinnao) | α-asarone from *Acorus tatarinowii* Schott | 0.5 mg/kg + 5 mL saline | 2 times/day | 5–7 |
| Tang et al. 2014 [31] | Oral Chinese Herbal Decoction (combined with Western nebulization) | *Morus alba* L. (*Sangbaipi*), *Lycium chinense* Mill. (*Digupi*), *Aster tataricus* L. f. (*Ziwan*), *Salvia miltiorrhiza* Bunge (*Danshen*), *Poria cocos* (Schwein.) F.A. Wolf (*Fuling*) 10 g each; *Scutellaria baicalensis* Georgi (*Huangqin*) 9 g; *Pinellia ternata* (Thunb.) Breitenb. (*Fabanxia*) 8 g; *Platycodon grandiflorus* (Jacq.) A.DC. (*Jiegeng*) 6 g; *Prunus armeniaca* L. (*Xingren*), *Fritillaria thunbergii* Miq. (*Zhebeimu*), *Citrus reticulata* Blanco (*Chenpi*) 5 g each; *Glycyrrhiza uralensis* Fisch. ex DC. (*Gancao*) 3 g. Modifications: For high fever, remove *Pinellia ternata* and add *Gypsum fibrosum* (*Sheng Shigao*; mineral) and *Phragmites australis* (Cav.) Trin. ex Steud. (*Shenglugen*); for thick yellow sputum, add *Trichosanthes kirilowii* Maxim. (*Gualoupi*) and bamboo sap (*Xianzhuli* from *Phyllostachys nigra* var. *henonis*); for blood-tinged sputum, add *Agrimonia pilosa* Ledeb. (*Xianhecao*); for severe wheezing, add *Pheretima aspergillum* (*Dilong*) and *Bombyx batryticatus* (*Jiangcan*); for chest pain, add *Citrus reticulata* vascular tissue (*Jieluo*); for low fever with red tongue and thready pulse, add *Artemisia annua* L. (*Qinghao*) and *Trichosanthes kirilowii* Maxim. (*Tianhuafen*) | One dose daily, decocted and taken orally in two divided doses (not nebulized) | Twice daily orally | Until cough resolves |
